# Low‐Resistance LiFePO_4_ Thick Film Electrode Processed with Dry Electrode Technology for High‐Energy‐Density Lithium‐Ion Batteries

**DOI:** 10.1002/smsc.202300302

**Published:** 2024-02-09

**Authors:** Kihwan Kwon, Jiwoon Kim, Seungmin Han, Joohyun Lee, Hyungjun Lee, Jiseok Kwon, Jungwoo Lee, Jihoon Seo, Patrick Joohyun Kim, Taeseup Song, Junghyun Choi

**Affiliations:** ^1^ Energy Storage Materials Center Korea Institute of Ceramic Engineering and Technology Jinju 52851 Republic of Korea; ^2^ Department of Applied Chemistry Kyungpook National University Daegu 41566 Republic of Korea; ^3^ Department of Energy Engineering Hanyang University 222 Wangsimni‐ro Seoul 04763 Republic of Korea; ^4^ Department of Materials Science and Engineering Pusan National University Pusan 46241 Republic of Korea; ^5^ Department of Chemical & Biomolecular Engineering Clarkson University Potsdam NY 13699 USA; ^6^ Department of Battery Engineering Gachon University 1342, Seongnam‐daero, Sujeong‐gu Seongnam‐si Gyeonggi‐do Republic of Korea

**Keywords:** dry process, lithium ion batteries, lithium iron phosphate, polytetrafluoroethylene

## Abstract

LiFePO_4_ emerges as a viable alternative to cobalt‐containing cathodes, such as Li[Ni_1–*x*–*y*_Mn_*x*_Co_*y*_]O_2_ and Li[Ni_1−*x*−*y*_Co_*x*_Al_*y*_]O_2_. As Fe is abundant in nature, LiFePO_4_ is a low‐cost material. Moreover, stable structure of LiFePO_4_ imparts long service life and thermal stability. However, the practical implementation of LiFePO_4_ cathode in energy storage devices is impeded by its low energy density and high ionic/electrical resistance. Herein, the LiFePO_4_ electrode with high active material loading and low ionic/electrical resistance through the dry process is reported for the first time. The dry process not only enables the uniform distribution of the polymeric binders and conductive additives within the thick electrode but also inhibits the formation of cracks. Furthermore, the bridge‐like connection of polytetrafluoroethylene facilitates the insertion and extraction of Li ions to the LiFePO_4_ crystal. Hence, the dry‐processed LiFePO_4_ electrode with high areal capacity (7.8 mAh cm^−2^) exhibits excellent cycle stability over 300 cycles in full‐cell operation. In addition, it is demonstrated that the estimated energy density of prismatic cell with the dry‐processed LiFePO_4_ electrode is competitive with state‐of‐the‐art Li[Ni_1–*x*–*y*_Mn_*x*_Co_*y*_]O_2_‐based battery.

## Graphical abstract

The dry process not only increases the active material loading but also improves the transport of Li ions within the electrode. Additionally, the dry process suppresses the side reactions of the electrolyte and stabilizes the cathode–electrolyte interphase layer. Owing to these advantages, the dry‐processed LiFePO_4_ (LFP) electrode outperforms the wet‐processed LFP electrode in terms of electrochemical performance.

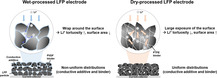

## 1 Introduction

In the last decade, the global electric vehicle (EV) count has experienced a remarkable surge, increasing from 17 000 to 7.2 million. With a projected compound annual growth rate of 30%, it is expected that the global EV count will approach 140 million by 2030.^[^ ^1^, ^2^ ^]^ Lithium‐ion batteries (LIBs) serve as the primary power source in EVs, and their performance indices are directly determined by the cathode material.^[^ ^3^, ^4^, ^5^ ^]^ LiFePO_4_ (LFP) is an ideal cathode material for LIBs due to its environmental sustainability, cost advantages, long service life, and thermal stability up to 270 °C.^[^ ^3^, ^6^ ^]^ In 2021, Tesla Inc. announced its decision to switch the cell chemistry in its mass‐market EVs from Li[Ni_1−*x*−*y*_Co_*x*_Al_*y*_]O_2_ (NCA) to LFP.^[^ ^7^, ^8^ ^]^ Despite their growing market share, LFP electrodes are still limited by their low electronic conductivity (10^−6^–10^−10^ S cm^−1^) and sluggish Li‐ion diffusion rates.^[^ ^6^, ^9^ ^]^ These adverse properties are attributed to the broken FeO_6_ networks in the LFP crystal structure, which hinder electron conduction and Li‐ion diffusion.^[^ ^6^ ^]^ Moreover, LFP suffers from a relatively low operating voltage (3.2 V vs Li/Li^+^) and theoretical specific capacity (≈170 mAh g^−1^),^[^ ^10^, ^11^, ^12^ ^]^ leading to lower energy density compared to Li[Ni_1–*x*–*y*_Mn_*x*_Co_*y*_]O_2_ (NMC) and NCA counterparts. Therefore, it is necessary to overcome the low energy density of LFP for practical applications.

**Figure 1:**
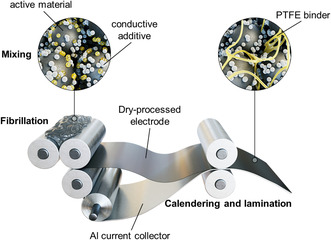
Schematic illustration of the dry electrode processes.

Among various efforts to improve energy density of LFP, constructing electrodes with a high loading of active materials is regarded as an effective approach because it can reduce the proportion of inactive components (e.g., separator, current collector, and packaging components) at the cell level.^[^ ^13^, ^14^ ^]^ Unfortunately, the conventional slurry‐based wet process causes severe issues in thick electrodes during the solvent evaporation step, such as crack formation and the nonuniform distribution of polymeric binders and conductive additives.^[^ ^15^ ^]^ These problems can adversely affect the mechanical and electrical properties of thick electrodes. In addition, *N*‐methyl‐2‐pyrrolidone (NMP), widely used as an organic solvent for mixing cathode composites in the wet process, is not only harmful to humans and the environment but also expensive due to the evaporation and recycling steps, which accounts for approximately 78% of the overall electrode production cost.^[^ ^16^, ^17^ ^]^ For these reasons, battery manufacturers have been striving to eliminate the use of NMP in the entire electrode production process. In this scenario, a dry process, which uses polytetrafluoroethylene (PTFE) as a deformable binder, has emerged as a potential method for fabricating thick electrodes without using any solvent. The dry process comprises three sequential steps (**Figure** **1**): 1) shear mixing electroactive particles, conductive additives, and the PTFE binder without solvent; 2) rolling and calendaring of the resulting mixture; and 3) laminating the electrode composites onto the current collector.^[^ ^18^ ^]^ Owing to the absence of solvent evaporation step, the dry process can substantially decrease battery manufacturing costs and easily construct electrodes with a high loading of active materials.^[^ ^15^, ^19^, ^20^ ^]^ Although the dry process is currently being extensively researched, the fabrication of an LFP thick electrode using this method has not been reported yet. Therefore, it is interesting to explore the enhanced energy density and electrochemical performance of an LFP electrode fabricated by the dry process.

In this article, we present an LFP electrode with a high areal capacity and low resistance achieved through the dry process. The dry process not only increases the active material loading but also improves the transport of Li ions within the electrode, thereby overcoming the inherent limitations of both the LFP material and electrode. Additionally, the dry process suppresses the side reactions of the electrolyte and stabilizes the cathode–electrolyte interphase (CEI) layer. Owing to the advantages of the dry process, the cell with the dry‐processed LFP electrode outperforms the that with the wet‐processed LFP electrode in terms of electrochemical performance. Most importantly, the dry‐processed LFP/graphite cell exhibits a high areal capacity (7.8 mAh cm^−2^), excellent gravimetric/volumetric energy density, and superior cycle stability even after 300 cycles. This study proposes a practical strategy for constructing electrodes to address issues related to the fabrication process and enhance the electrochemical performance.

## 2 Results and Discussion

**Figure 2:**
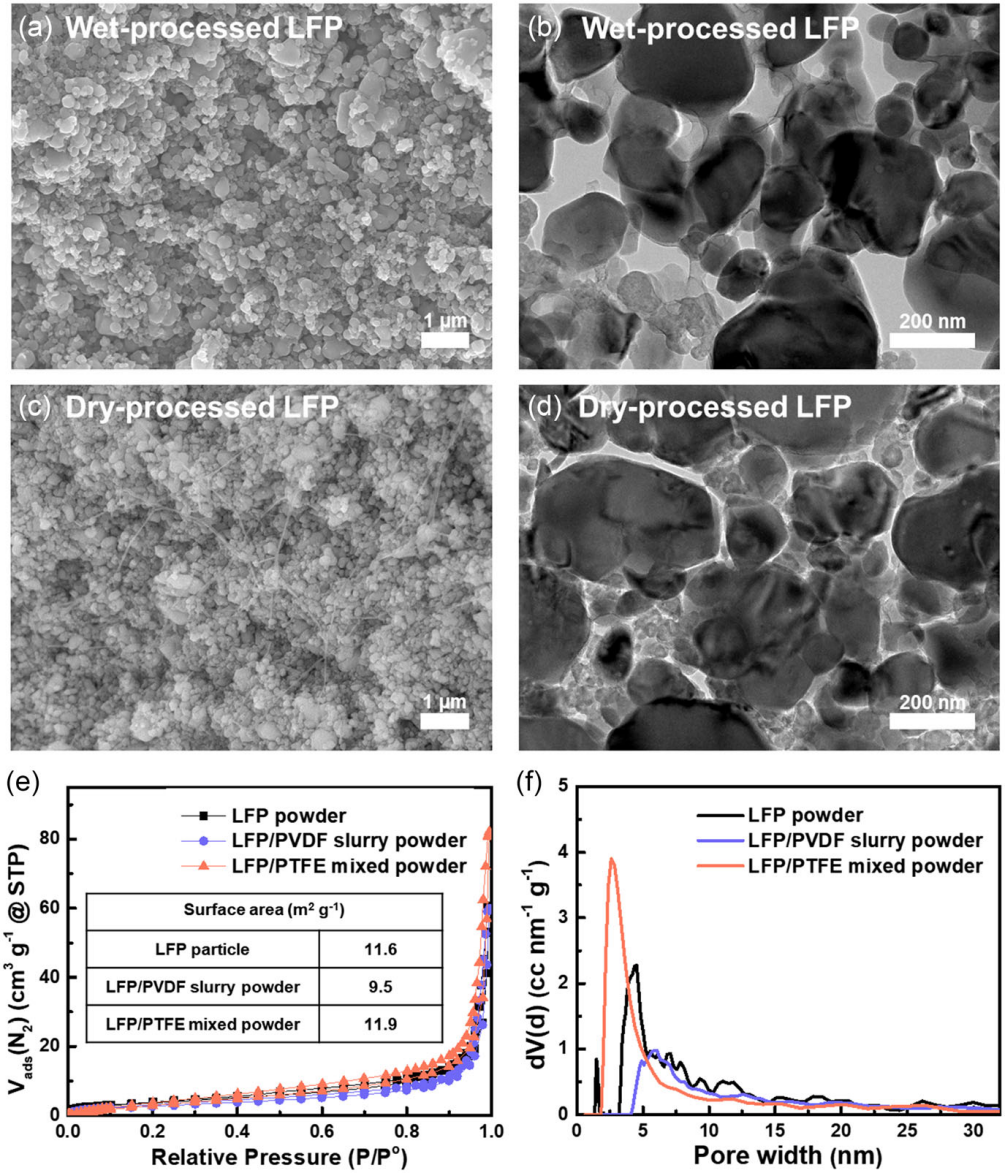
Characterization of binders within the LFP electrodes. a,b) Cross‐sectional SEM and Cs‐TEM images of the wet‐processed LFP electrode. c,d) Cross‐sectional SEM and Cs‐TEM images of the dry‐processed LFP electrode. e) Nitrogen adsorption–desorption isotherms and f) pore size distribution curves of wet‐ and dry‐processed LFP electrodes obtained using BET measurements.

**Figure** **2** shows the morphologies and characteristics of the wet‐ and dry‐processed LFP electrodes. The morphologies of the binders within the LFP electrodes were compared using cross‐sectional SEM (Figure 2a,c). The fiberized PTFE binder can be observed in the dry‐processed LFP electrode. The bridge‐like connection of fibrous PTFE enables efficient Li‐ion diffusion into the LFP crystal in the dry‐processed electrode. To further understand the binding features, both the wet‐ and dry‐processed LFP electrodes were analyzed using Cs‐TEM after FIB milling (Figure 2b,d). In the wet‐processed LFP electrode, the adhesive binder, i.e., PVDF, covers the surfaces of the LFP particles. Notably, the diffusion of Li ions in LFP occurs unidirectionally along the *b*‐axis.^[^ ^3^, ^21^ ^]^ Therefore, the PVDF binder can considerably hinder the extraction and insertion of Li ions in olivine‐based LFP electrodes. The specific surface areas and pore size distributions of particles in the electrode were measured using nitrogen adsorption–desorption isotherms (Figure 2e,f). The specific surface area of the LFP/PTFE mixed powder (11.9 m^2^ g^−1^) is equivalent to that of the LFP powder (11.6 m^2^ g^−1^). This result can be attributed to the spot contact between the fiberized PTFE and LFP particles. In contrast, the LFP/PVDF slurry powder has a relatively lower number of micropores and a lower specific surface area (9.5 m^2^ g^−1^). This is because the PVDF binder covers the surfaces of the LFP particles. These disadvantages remarkably decrease the diffusion efficiency of Li ions.

**Figure 3:**
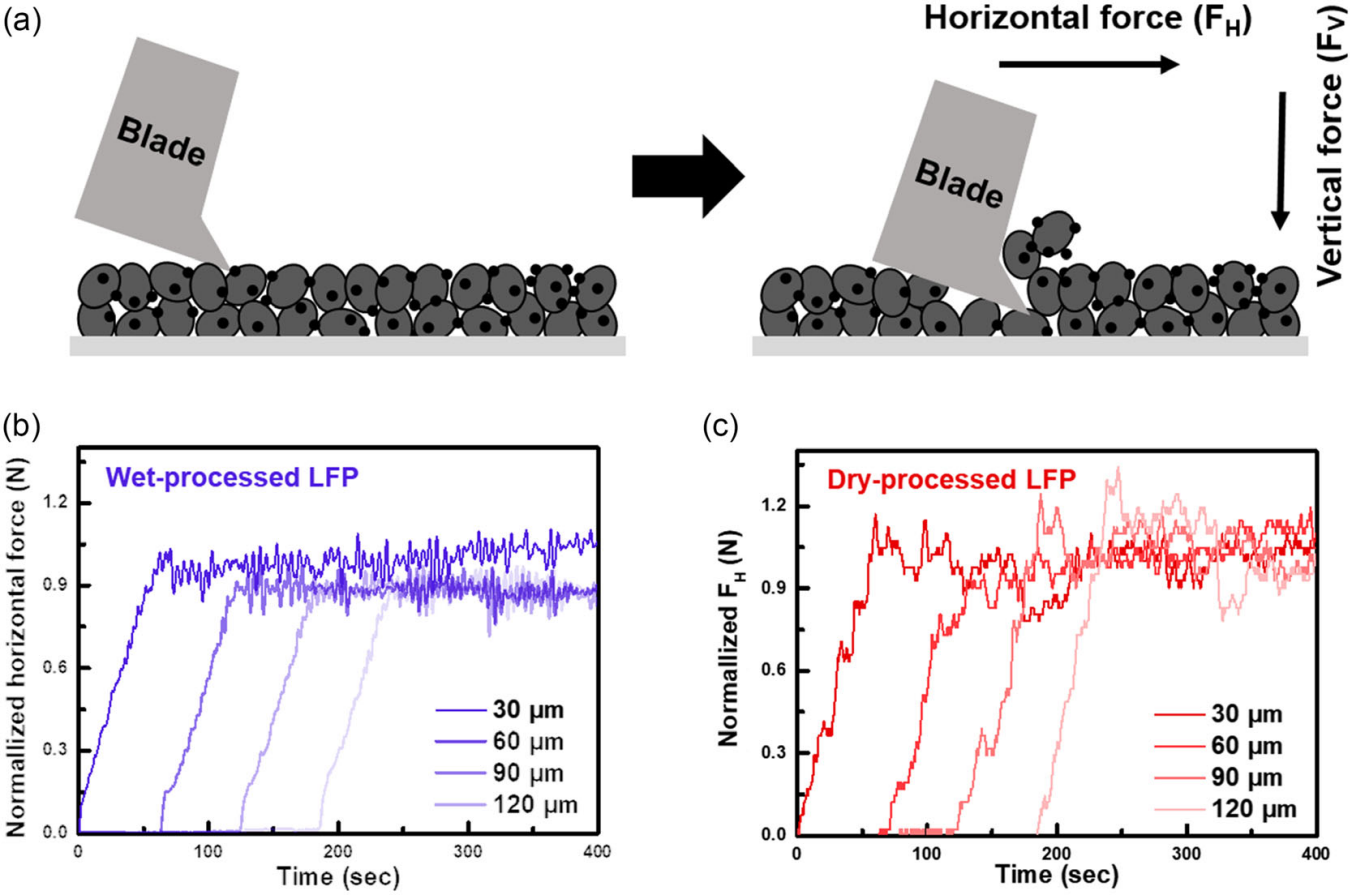
SAICAS analysis of LFP electrode. a) Schematic illustration of the SAICAS to examine the adhesion strength according to the depth from the electrode surface. b,c) The normalized *F*_H_ at every 30 μm of depth from the surfaces of wet‐ and dry‐processed LFP electrode.

To examine the dispersion of the polymeric binder within each electrode, the adhesion strengths, which are proportional to the binder ratio, were measured by using the SAICAS at every 30 μm of depth from the electrode surfaces (**Figure** **3**). In the conventional peel test, adhesion strengths are measured by placing adhesive tape on the surface of the sample and obtaining the force during pulling. However, the peel test is inadequate for examining the binder dispersion within electrodes because the weakest interface is consistently detached preferentially. In contrast to the peel test, SAICAS can offer sufficient adhesive strength at a specific depth or location within electrodes. Figure 3a shows a schematic illustration of the SAICAS test. As the SAICAS employs an angled blade, the horizontal (*F*_H_) and vertical forces (*F*_V_) can be measured along the surface.^[^ ^22^ ^]^ During consecutive passes of the slanted blade, the two perpendicular forces, i.e., *F*_H_ and *F*_V_, are used simultaneously to moderately cut the electrode at a specific depth, and the adhesion strength at that location is evaluated using the *F*_H_. The binder distribution at a specific depth of the electrode can be estimated from the *F*_H_ fluctuation profiles. As shown in the *F*_H_ profiles (Figure 3b,c), the dry‐processed LFP electrode exhibits nearly similar adhesion strengths within the electrode. This observation indicates that the binder is uniformly distributed within the dry‐processed electrode. In contrast, the adhesion strength of the wet‐processed LFP electrode decreases (by approximately 12%) as the depth increases from the top to the middle and bottom points. This result is closely related to the binder migration near the surface of the electrode during solvent evaporation. This adversely affects the mechanical and electrical properties and the Li‐ion transport kinetics of the wet‐processed LFP electrode.

**Figure 4:**
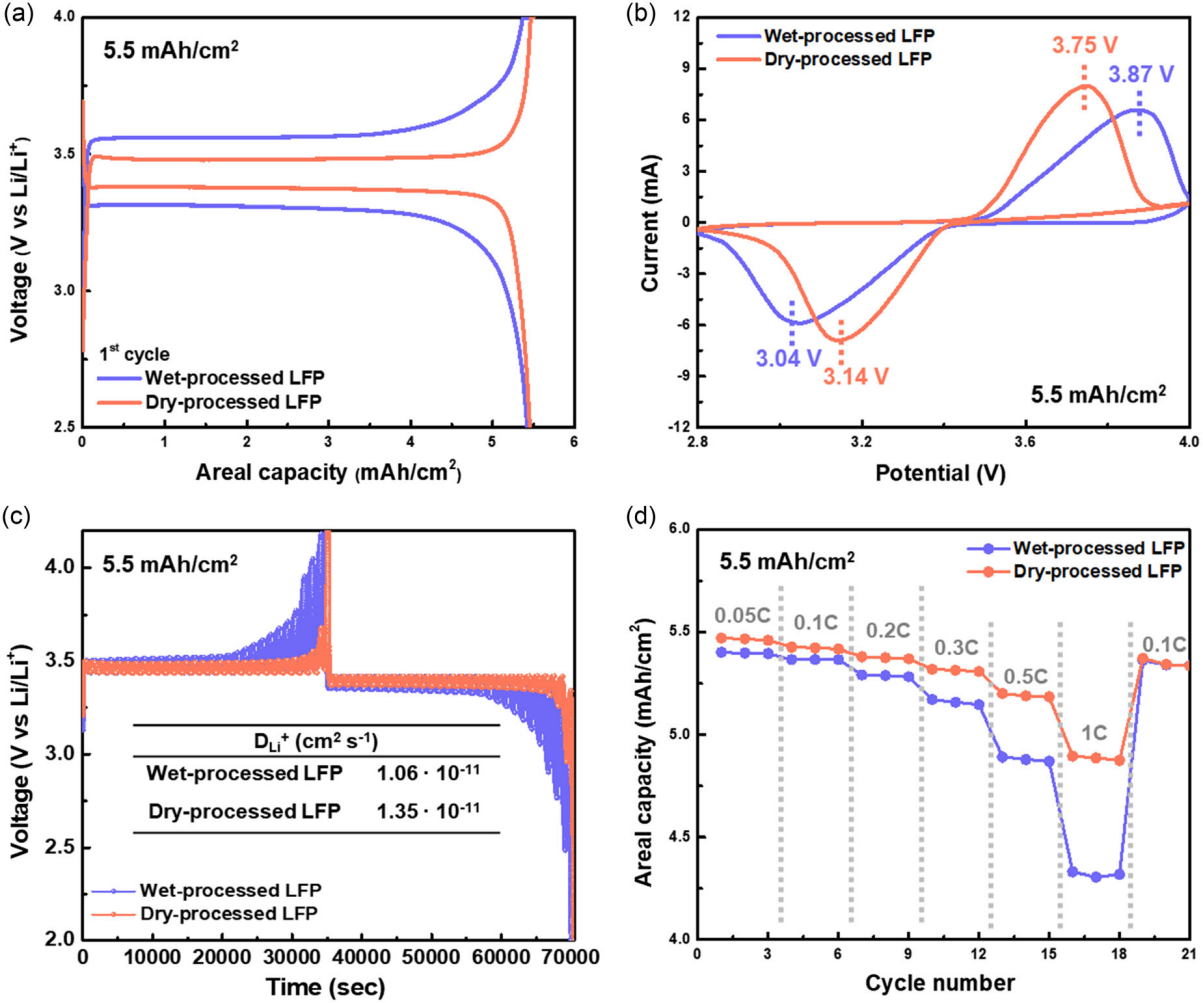
Electrochemical performances of LFP‐Li cells with the wet‐ and dry‐processed LFP electrodes. a) The voltage profiles of the first cycle and b) the CV measurement at 1 mV s^−1^. c) The calculated *D*_Li_^+^ from the GITT analysis. d) Rate capability from 0.05 to 1C.

The LFP‐Li cells were fabricated to evaluate the electrochemical properties of the wet‐ and dry‐processed LFP electrodes with the same areal capacity of 5.5 mAh cm^−2^ (35 mg cm^−2^). In the first cycle voltage profile, the dry‐processed electrode exhibits a low‐voltage hysteresis (Δ*V*) between the lithiation and delithiation in the LFP electrodes, which is associated with low overpotential during charging and discharging (**Figure** **4**a).^[^ ^23^, ^24^ ^]^ Unlike the dry‐processed electrode, a large internal resistance drop is observed in the wet‐processed electrode during the initial charging phase of the GITT (Figure S1, Supporting Information).^[^ ^25^ ^]^ The large potential difference in the wet‐processed electrode is originated from the large resistance within the electrode, which may have resulted from the presence of the PVDF binder covering a large surface area of the LFP particles. The PVDF binder coverage of the LFP particles hinders the insertion and extraction of Li ions from LFP. The dry‐processed electrode with low resistance exhibits an enhanced cycle stability at 0.2C, whereas the wet‐processed electrode experiences severe discharge capacity degradation after only five cycles (Figure S2, Supporting Information). To demonstrate the consistency of the electrochemical performance, Table S1, Supporting Information provides information on the all tested LFP‐Li cells.

CV measurement was performed at a scan rate of 1 mV s^−1^ to validate the low‐voltage hysteresis (Δ*V*) of the dry‐processed electrode,^[^ ^26^ ^]^ which shows a 26% lower voltage hysteresis than the wet‐processed one (0.83); these values correspond to the behavior of the electrodes in the first cycle voltage profile (Figure 4b). The Li‐ion diffusion coefficient (*D*_Li_^+^) of the dry‐processed electrode was calculated to be 1.35 × 10^−11^ cm^2^ s^−1^, which is 27.3% higher than that of the wet‐processed electrode (1.06 × 10^−11^ cm^2^ s^−1^); the *D*_Li_^+^ values were calculated using GITT (Figure 4c and S3, Supporting Information).^[^ ^27^ ^]^ The spot contact between the PTFE binder and LFP, attributed to the fibrous morphology of the PTFE binder, improves the insertion and extraction of Li ions from LFP and lowers the resistance within electrode, resulting in the high *D*_Li_^+^ values.

The rate capability test was further conducted to observe the influence of Li‐ion diffusion on the electrochemical properties within the electrode. The dry‐processed electrode shows a larger discharge capacity than the wet‐processed electrode as the C‐rate increased from 0.05–1C. At 1C, the retention of the discharge capacity for the wet‐ and dry‐processed electrodes is 89% and 80%, respectively, relative to the capacity at 0.05C (Figure 4d and S4, Supporting Information). The high diffusion of Li ions within the dry‐processed electrode is considered to contribute to its enhanced electrochemical properties,^[^ ^28^ ^]^ which results from the fibrous morphology of PTFE. Moreover, the spot contact between the binder and LFP lowers the ionic resistance within the electrode. The comparative evaluation of the electrochemical properties of both electrodes suggests that the diffusion of Li ions within the electrode is closely related to the resistance within the electrode.^[^ ^29^ ^]^ The resistance is, in turn, associated with the morphology and distribution of the polymeric binders (PTFE and PVDF) near the surface of LFP electrodes.

**Figure 5:**
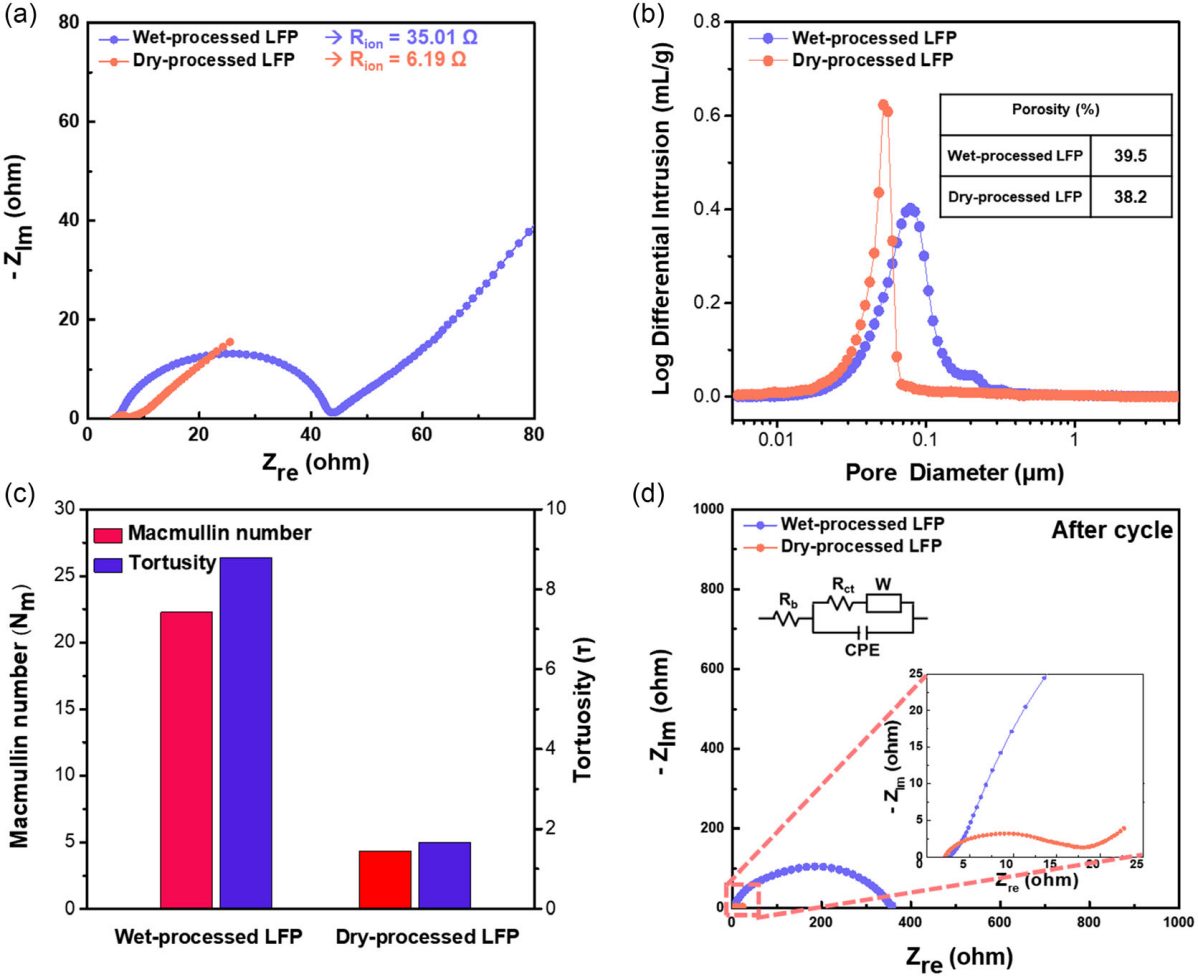
Investigation of microstructure of wet‐ and dry‐processed LFP electrodes. a) The EIS spectra of each symmetric cell before cycle for the evaluation of *R*_ion_ and b) pore distribution of each electrode by mercury porosimetry. c) The MacMullin number (*N*_m_) and tortuosity (*τ*) of each electrode, related with Li‐ion transport through pore channel within electrode. d) The EIS spectra of each LFP‐Li cell after cycle for the evaluation of *R*_ct_.

The uniform distribution of the binder throughout the electrode is a critical factor in reducing the electrode resistance.^[^ ^30^, ^31^ ^]^ As briefly mentioned before, the wet process involves a solvent evaporation step that can lead to binder migration; this results in the nonuniform distribution of the PVDF binder and poor electrochemical properties. In contrast, the dry process does not involve a solvent evaporation step and results in the uniform distribution of the PTFE binder and enhances the electrochemical properties of the electrode.^[^ ^19^ ^]^ Symmetric cells were fabricated to investigate the uniformity of the microstructures in both wet‐ and dry‐processed electrodes. In addition, the *R*_ion_ values of the electrodes obtained using EIS were compared. *R*_ion_ is directly related to the Li‐ion transport through the pore channels within the electrode (**Figure** **5**a).^[^ ^32^ ^]^ The dry‐processed electrode exhibits an 83% reduction in the *R*_ion_ value compared to the wet‐processed electrode. This result indicates that the uniform distribution of PTFE improves the Li‐ion transport through the pore channels by enhancing the uniformity of the electrode microstructure. To confirm the uniformity of the microstructure, mercury porosimetry was carried out (Figure S5, Supporting Information).^[^ ^33^ ^]^ The porosity of the dry‐processed electrode (38.2%) is lower than that of the wet‐processed electrode (39.5%) owing to the low density of the wet‐processed electrode (Figure 5b). A high‐density wet‐processed electrode could not be fabricated at high areal capacity (>2.2 g cc^−1^, >5 mA h cm^−2^) because of delamination between electrode layer and current collector. In addition, the average pore size of the dry‐processed electrode was lower than that of the wet‐processed electrode. In terms of pore distribution, the dry‐processed electrode displays a narrower peak width in the range of 0.02–0.1 μm compared to the wet‐processed electrode, which exhibits a broad peak width in the range of 0.02–0.3 μm. The narrower peak width indicates the uniform pore size and uniform microstructure of the dry‐processed electrode. The uniform microstructure of the dry‐processed electrode can be attributed to its uniform binder distribution, which is associated with the absence of binder migration during the solvent evaporation process.^[^ ^34^ ^]^ This considerably enhances the Li‐ion transport through the pore channels, the *R*_ion_ values, and ultimately the electrochemical performance of the electrode. The improved resistance to Li‐ion transport within the dry‐processed electrode can be demonstrated by its MacMullin number (*N*_m_) and tortuosity (τ); these parameters represent Li ions transport through the pore channels, regardless of the thickness and porosity of the electrode (Figure 5c).^[^ ^35^, ^36^ ^]^ Both the *N*_m_ and *τ* values of the dry‐processed electrode were lower than those of the wet‐processed electrode, despite its low thickness and porosity. The low *N*_m_ and *τ* values reveal that even with high electrode density and low porosity, the dry‐processed electrode exhibits low resistance to Li ions transport through the pore channels because of its uniform microstructure. To investigate the resistance of charge transfer (*R*_ct_) during cell operation, EIS measurements were carried out both before and after cycling. Prior to cycling, the *R*_ct_ values of the wet‐ and dry‐processed electrodes are similar (Figure S6, Supporting Information). After cycling, the *R*_ct_ value of the dry‐processed electrode is lower than that of the wet‐processed electrode (Figure 5d). It is highly likely that the *R*_ct_ value of the dry‐processed electrode is lower because of the lower resistance to Li‐ion transport through the pore channels.^[^ ^37^ ^]^ After cycling, the reduction in the *R*_ct_ value is unexpectedly large, and the LFP surface was further analyzed for comprehensive understanding. XPS was conducted before and after cycling to investigate the effects of binder morphology and distribution on *R*_ct_ (Figure S7, Supporting Information). The F 1s spectra provide valuable information about the composition of the CEI of the LFP electrode, particularly allowing for a relative comparison of LiF formation resulting from the decomposition of LiPF_6_ salt in the electrolyte.^[^ ^17^ ^]^ Before cycling, only peaks corresponding to PVDF (687.8 eV) and PTFE (689.9 eV) were detected (Figure S7a,b, Supporting Information, respectively). After cycling, the F 1s spectra of the wet‐processed electrode show higher abundance of LiF than that of the dry‐processed electrode (Figure S7c,d, Supporting Information). The evaluation of the ratios of LiF to polymeric binders (PTFE and PVDF) reveals that the dry‐processed electrode (which has low binder coverage on the surface of the LFP electrode and a low *R*_ion_ value) may reduce LiPF_6_ decomposition and LiF formation, thereby forming a homogeneous CEI.^[^ ^38^ ^]^ The homogeneous CEI lowers the *R*_ct_ after cycling, which results in superior electrochemical properties. It would be attributed to the fact that the distribution of polymeric binders or conductive agents near the surface of active materials has a significant impact on electrochemical performance. The distribution of these components significantly influences the kinetics of Li ions, associated with the insertion and extraction of Li ions from the active materials during the charge and discharge.^[^ ^39^ ^]^ Specifically, when the ion or electrical conductivity of the surface of active materials is poor, it detrimentally affects the kinetics of Li ions, inducing the surface side reaction and the formation of a thick CEI layer on the surface of active materials during the cell operation.^[^ ^40^ ^]^ The formation of thick CEI layer can increase the interfacial resistance of active materials, contributing to the electrode degradation and low coulombic efficiency.^[^ ^38^ ^]^ Additionally, both PVDF and PTFE within the electrode remain unreacted after cycling, indicating that the formation of LiF is not related to the electrochemical reactions of binders. A previous study reported that PVDF is stable within the voltage range of LFP operation.^[^ ^41^ ^]^ Similarly, the stability of PTFE within the operational voltage range of LFP was observed by the presence of the CF_2_ peak, which corresponds to PTFE both before and after cycling (Figure S8, Supporting Information). XRD results indicate a persistent presence of the PTFE peak at 18° both before and after cycling, which aligns with the XPS results (Figure S9, Supporting Information). The presence of unreacted polymeric binders (PVDF and PTFE) indicates that the formation of LiF solely depends on the decomposition of LiPF_6_ and the solvent in the electrolyte, and the correlation between LiF formation and *R*_ct_ is substantiated. To further investigate the cycle stability of each electrode, cross‐sectional SEM images were obtained after cycling (Figure S10, Supporting Information). Unlike the dry‐processed LFP electrode, the cracks of LFP particles in the wet‐processed LFP electrode were observed, as shown in Figure S10a, Supporting Information. It would be attributed to the high stress of inserting and extracting Li ions in the particles of the wet‐processed LFP electrode.^[^ ^42^ ^]^

**Figure 6:**
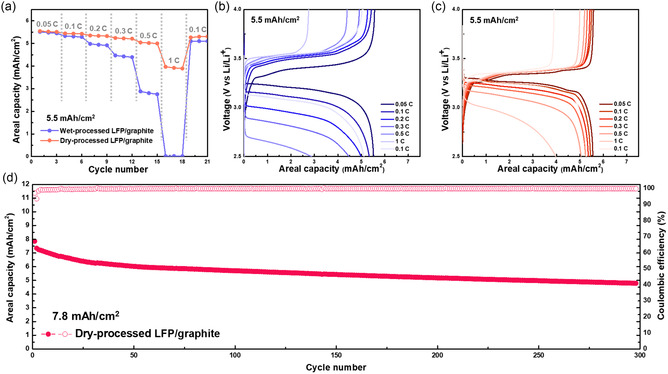
Electrochemical performance of LFP/graphite cell. a) The rate capability and b,c) voltage profiles from 0.05 to 1C of b) wet‐ and c) dry‐processed LFP/graphite cell (5.5 mAh cm^−2^ and N/P ratio: 1.1). d) The cycle performance of dry‐processed LFP/graphite cell with 7.8 mAh cm^−2^ at 2.4 mA cm^−2^.

To compare the electrochemical kinetics of the wet‐ and dry‐processed LFP electrodes, each full cell with high areal capacities (5.5 mAh cm^−2^, N/P ratio: 1.1) was evaluated under different current conditions. The wet‐processed LFP/graphite cell shows a serious degradation of discharge capacity as C‐rate increases. It almost reaches near‐zero capacity when it was tested at 1C (**Figure** **6**a,b). In contrast, the dry‐processed LFP/graphite cell shows a discharge capacity of 4 mAh cm^−2^ at 1C; this value was approximately 75% of its discharge capacity at 0.05C (Figure 6c). As previously discussed, low resistance to Li ions transport through the pore channel and charge transfer occur because of the uniform microstructure and homogeneous CEI of the LFP. This feature leads to high Li‐ion diffusion within the dry‐processed electrode, thereby enhancing the rate capability of the dry‐processed full cell. Based on these advantages of the dry‐processed electrode, a dry‐processed LFP/graphite cell with a much higher areal capacity (7.8 mAh cm^−2^) and electrode density (2.3 g cc^−1^) was successfully fabricated. However, it is technically difficult to fabricate a wet‐processed electrode with the same areal capacity and electrode density because of issues related to crack formation (Figure S11, Supporting Information). The crack formation issues revealed that the wet‐processed electrode exhibits weak adhesion strength between the electrode components owing to the nonuniform distribution of the PVDF binder.^[^ ^38^ ^]^ The nonuniform distribution of the binder is associated with binder migration during the solvent evaporation process. The dry‐processed LFP/graphite full cell with an areal capacity of 7.8 mAh cm^−2^ achieved excellent cycle stability over 300 cycles (Figure 6d and S12, Supporting Information). Most of the previous studies with regard to LFP full cells have evaluated LFP electrodes with low areal capacity (≈3 mAh cm^−2^) and low density (≈2.1 g cc^−1^).

**Figure 7:**
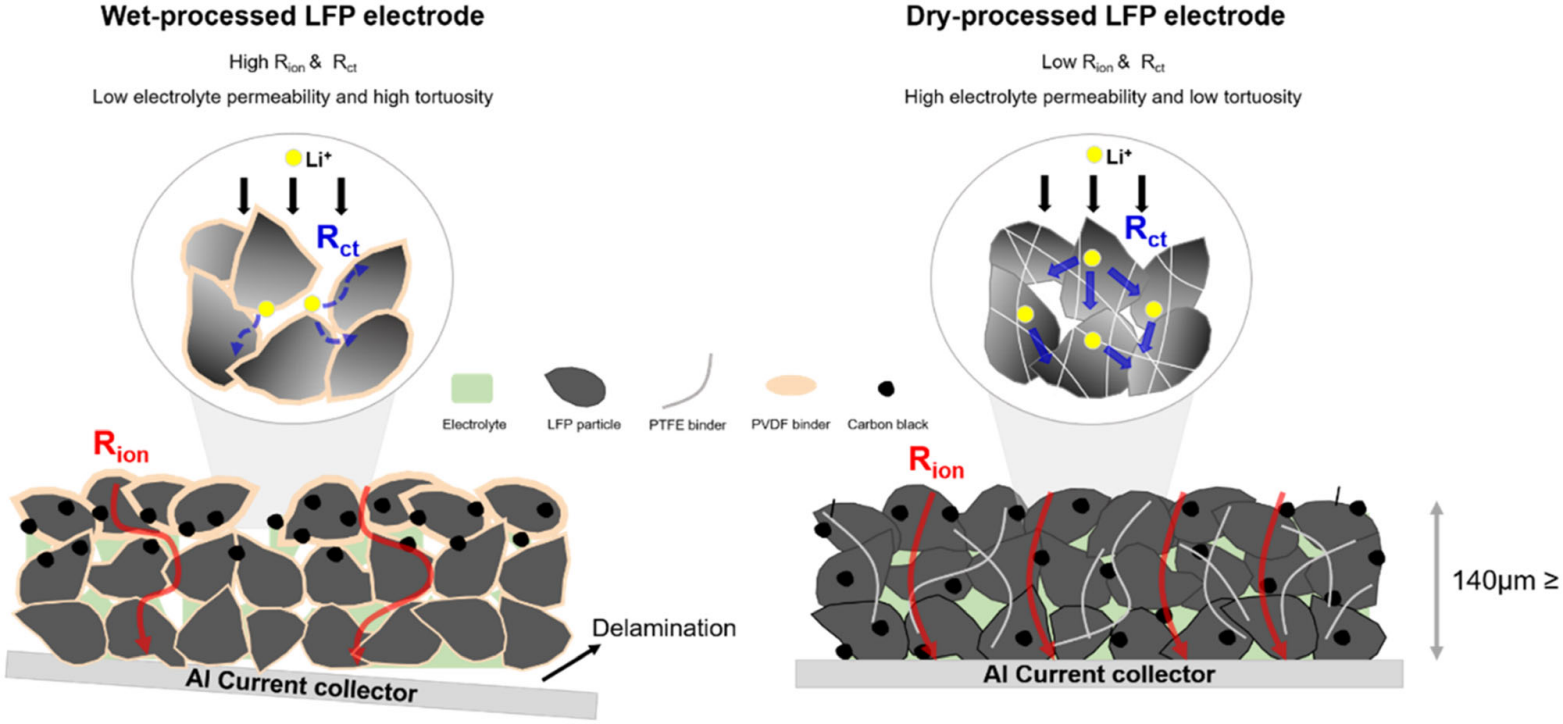
Schematic illustration. The morphology of polymeric binder and distributions of conductive additives and polymeric binders in wet‐ and dry‐processed LFP electrode.

**Figure 8:**
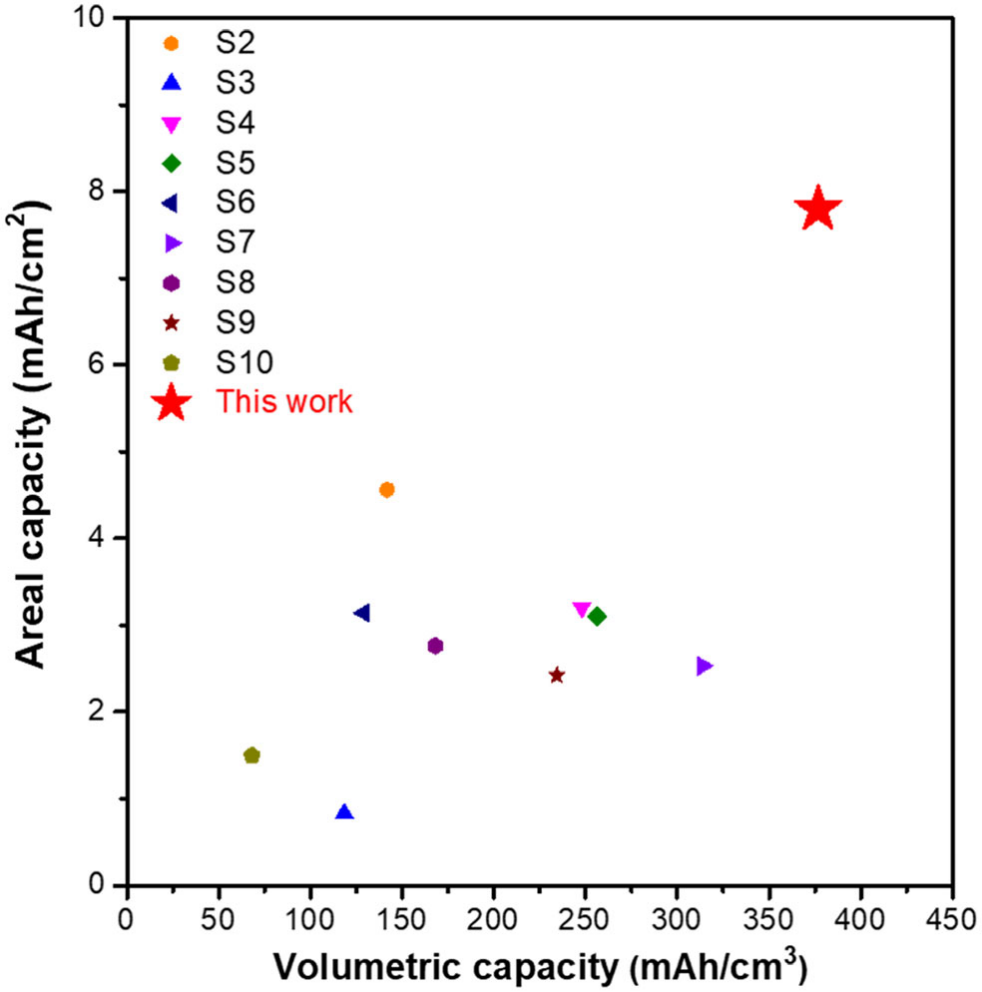
The dry‐processed LFP/graphite cell with high areal capacity. The areal capacity of LFP electrode as a function of volumetric capacity (this work vs previously reported LFP electrode).

The high energy density cathode prepared through the dry process has several advantages over the wet process. **Figure** **7** summarizes the distinct mechanisms of Li‐ion transport within wet‐ and dry‐processed LFP electrodes. As the slurry‐based wet process involves the solvent evaporation step, low‐density inactive materials, such as the PVDF binder and conductive additive, migrate and agglomerate near the electrode surface owing to capillary force. This phenomenon aggravates the wettability of the electrolyte and thus impedes the diffusion of Li ions through the electrode microstructure.^[^ ^30^, ^34^, ^43^ ^]^ Moreover, the PVDF binder wraps around the surface of active material, thereby reducing the sites for the insertion and extraction of Li ions. In the dry‐processed LFP electrode, the absence of the solvent evaporation step enables the uniform dispersion of inactive materials within the electrode. In addition, bridge‐like connection of PTFE exposes the active material surface. These advantages of the dry process not only reduce the ionic resistance (*R*_ion_) associated with Li‐ion tortuosity but also decrease the charge transfer resistance (*R*_ct_) for the insertion and extraction of Li ions in LFP crystals. Due to these synergistic benefits, the dry‐processed LFP/graphite cell has achieved high volumetric capacity of over 370 mA h cm^−3^. The volumetric capacity is higher than those reported in previous studies (**Figure** **8**, Table S2, Supporting Information).

To evaluate the practical impact of our work, the estimated energy densities of prismatic cells are calculated based upon the previous reports and are summarized in Table S3, Supporting Information. As crack formation and delamination from the current collector occur during the wet process, it is tricky to construct the wet‐processed LFP electrode with an areal capacity above 5.0 mAh cm^−2^. Therefore, the maximum gravimetric/volumetric energy density of the wet‐processed LFP electrode is limited to 153 Wh kg^−1^/390 Wh L^−1^. Meanwhile, the gravimetric/volumetric energy density of the dry‐processed LFP electrode can be maximized to 185 Wh kg^−1^/470 Wh L^−1^ owing to its advantage of fabrication process. Especially, when the dry process is integrated with the cell‐to‐pack (CTP) technology, it can exhibit a synergistic effect on energy density. The CTP technology, which directly assembles cells into packs, enables the elimination of module components; this process can greatly increase the gravimetric and volumetric energy densities by 10–15% and 50%, respectively.^[^ ^44^ ^]^ When the CTP technology is applied to the dry‐processed LFP‐based battery cell, it can achieve a gravimetric/volumetric energy density of 213 Wh kg^−1^/705 Wh L^−1^, making it competitive with state‐of‐the‐art NMC‐based battery.

## 3 Conclusion

In this study, an LFP electrode with a high proportion of active materials and high areal capacity was successfully constructed using the binder fibrillation process. The electrochemical performance of the LFP‐Li cell using the dry‐processed LFP electrode was substantially higher than that using the wet‐processed LFP electrode. The superior performance of the dry‐processed electrode can be ascribed to the following properties: 1) homogeneous dispersion of the conductive additive and polymeric binder in the thick electrode; 2) binding of electrode composite materials by spot contact with the PTFE binder; and 3) generation of fewer F‐containing CEI components, which leads to excellent interfacial stability. Owing to these synergistic benefits, the dry‐processed LFP electrode exhibited a high areal capacity (7.8 mA h cm^−2^) and stable cycle performance after 300 cycles in a full cell coupled with a graphite anode. This study opens new avenues for the advancement of dry‐processed LFP electrode fabrication for LIBs with higher energy density and low ionic/electrical resistance.

## 4 Experimental Section

4.1

4.1.1

##### Preparation of the Electrodes

A slurry was prepared by blending LFP (LFP‐NCO, Advanced Lithium Electrochemistry Co. Ltd.), carbon black (Super P), and polyvinylidene fluoride (PVDF, Solef 5130, *M*_w_ = 1 100 000 g mol^−1^) in NMP (DAEJUNG, purity >99.7%) to fabricate the wet‐processed LFP electrode. The slurry was then tape‐cast onto a 20 μm thick Al current collector and dried overnight in a vacuum oven at 100 °C. The dry‐processed LFP mixture was produced by mixing LFP, carbon black (Super P), and the PTFE binder (F‐104, DAIKIN) using a planetary mixer (ARE‐310, THINKY) and a pestle. The resultant mixture was then pressed and laminated onto a 20 μm thick Al current collector at 60 °C. The mass ratio of LFP:Super P:binder was 97:1:2 in both wet‐ and dry‐processed LFP electrodes. Through the calendaring process to increase the electrode density and tailor the microstructure, the electrode densities of the wet‐ and dry‐processed LFP electrodes were set to 2.1 and 2.3 g cc^−1^, respectively. And, the thickness of the wet‐ and dry‐processed LFP electrodes was 190 and 175 μm, respectively.

##### Characterization

Field emission scanning electron microscopy (FE‐SEM, MIRA II LMH, TESCAN) and Cs‐corrected transmission electron microscopy (Cs‐TEM, Titan Themis Z, Thermo Scientific) were used to investigate the morphologies and microstructures of the wet‐ and dry‐processed LFP electrodes. Focused ion beam (FIB, Thermo Scientific, Brno) milling was performed to prepare the samples for Cs‐TEM analysis. X‐ray diffraction (XRD) measurements of the PTFE and LFP particles were carried out with the D8 Advance instrument. Surface areas and pore distributions were characterized using a surface area and pore size analyzer Brunauer–Emmett–Teller (BET, Quadrasorb evo, Quantachrome). Surface and interfacial cutting analysis system (SAICAS, SAICAS EN‐EX, Daipla Wintes) was employed with a diamond blade (width = 1 mm, shear angle = 45°, rake angle = 20°, and clearance angle = 10°) to evaluate the adhesion strengths at different depths from the electrode surface. The composition of CEI layer on each LFP cathode was identified by X‐ray photoelectron spectroscopy (XPS, NEXSA, ThermoFisher). Mercury porosimetry techniques (Autopore v, Micromeritics) were used to measure the pore size distribution of the LFP cathodes.

##### Cell Assembly and Electrochemical Measurements

The coin‐type cells (CR2032, Wellcos Corporation) were assembled in a dry room with a dewpoint below −50 °C. In the half‐cells, a polypropylene separator (Celgard 3501, CELGARD), 1.15 M LiPF_6_ in a mixture of ethylene carbonate, ethyl methyl carbonate, and dimethyl carbonate (EC:EMC:DMC = 2:4:4, v/v/v) with 1 wt% LiPO_2_F_2_ and 1 wt% vinylene carbonate additive and 1.0 T Li‐metal foil (utilized as counter electrode) were used to examine the electrochemical performance of each LFP electrode (5.5 mA h cm^−2^, 35 mg cm^−2^). Galvanostatic charge/discharge tests were conducted under the constant current–constant voltage (CC–CV) protocol using a battery cycler (WBCS3000L32, WonATech). Prior to the cycle tests, two precycles at 0.05 and 0.1C (1C = 5.5 mAh cm^−2^) were carried out as a formation step. Except the charge/discharge at 0.05C, the discharge rate capability was evaluated at diverse current rates of 0.1, 0.2, 0.3, 0.5, and 1C after charging at a fixed current of 0.1C. Cyclic voltammetry (CV) measurements were performed at a constant scan rate of 0.1 mV s^−1^ with a voltage window of 2.8–4.0 V (VSP, Biologic). Electrochemical impedance spectroscopy (EIS, VSP, Biologic) was recorded at frequency range from 250 kHZ to 10 MHz with an amplitude of 5 mV. Galvanostatic intermittent titration technique (GITT, WBCS3000L32, WonATech) was conducted to calculate the Li‐ion diffusion coefficients by applying a pulse current of 0.1 mA cm^−2^ for a duration of 10 min, followed by pause times of 30 min in a voltage window of 2.0–4.2 V (vs Li/Li^+^). In the full‐cell configuration, the LFP cathodes were coupled with graphite anodes (graphite:Super P:binder = 96:1:3) at an N/P ratio of 1.1. Over the voltage window of 2.5–4.0 V versus Li/Li^+^, the cycle performance of full cells was tested at a rate of 0.2C after two formation steps at 0.05 and 0.1C. Symmetric pouch cells were assembled to measure the Li‐ion transfer resistance (*R*_ion_) of the electrodes. The *R*_ion_ was calculated in the frequency range of 1–1 MHz with an amplitude of 10 mV.

## Conflict of Interest

The authors declare no conflict of interest.

## Supporting information

Supplementary Material

## Data Availability

The data that support the findings of this study are available from the corresponding author upon reasonable request.
