# Supplementary material for: Low‐Resistance LiFePO4 Thick Film Electrode Processed with Dry Electrode Technology for High‐Energy‐Density Lithium‐Ion Batteries
Source: Small Sci. 2024 Feb 9;4(5):2300302. doi: 10.1002/smsc.202300302 (PMC11934976; doi:10.1002/smsc.202300302)
Supplement: Supplementary file 1 — Supplementary Material [file SMSC-4-2300302-s001.pdf]

[Supplementary information]

Low-Resistance $LiFePO_4$ Thick Film Electrode Processed with Dry Electrode Technology for High-Energy-Density Lithium-Ion Batteries

Kihwan Kwon[a,b],‡, Jiwoon Kim[c],‡, Seungmin Han[a,c], Joohyun Lee[a,d], Hyungjun Lee[c], Jiseok Kwon[c], Jungwoo Lee[d], Jihoon Seo[e], Patrick Joohyun Kim[b,] , Taeseup Song[c, *] and Junghyun Choi[a,f]**

[a] Energy Storage Materials Center, Korea Institute of Ceramic Engineering and Technology, Jinju, 52851, Republic of Korea

[b] Department of Applied Chemistry, Kyungpook National University, Daegu, 41566, Republic of Korea; pjkim@knu.ac.kr

[c] Department of Energy Engineering, Hanyang University, 222 Wangsimni-ro, Seoul 04763, Republic of Korea; tssong@hanyang.ac.kr

[d] Department of Materials Science and Engineering, Pusan National University, Pusan 46241, Republic of Korea

[e] Department of Chemical & Biomolecular Eng, Clarkson University, Potsdam, NY-13699

[f] Department of Battery Engineering, Gachon University, 1342, Seongnam-daero, Sujeong-gu, Seongnam-si, Gyeonggi-do, Republic of Korea; junghchoi@gachon.ac.kr

‡Equally contributed authors

*Corresponding authors

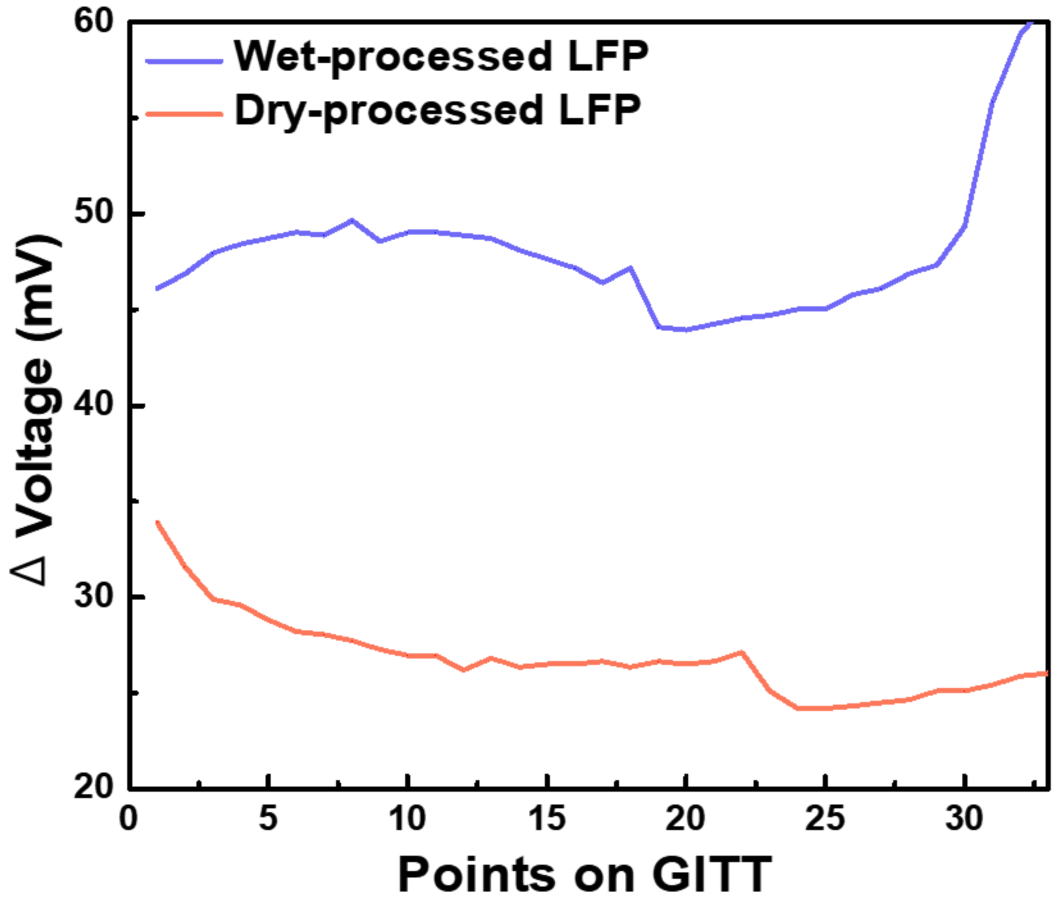

Figure S1. The IR drop of wet- and dry-processed LFP electrode from GITT analysis.

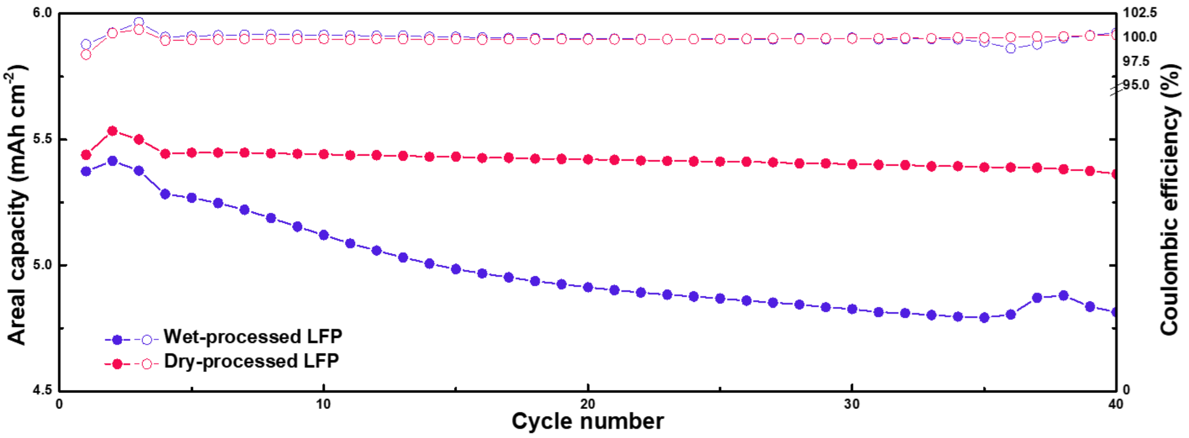

Figure S2. The cycle performances of wet- and dry-processed LFP-Li cells at 1.08 mA cm^{-2}.

	L.oading Level	Cathode Current Density	Electrode Density	1st cycle		40th cycle		Capacity Retention Rate after 40 cycle
				Capacity of charge/discharge	ICE	Capacity of charge/discharge	C.E.	
**Wet 1**	34.69 mg/cm^2	5.48 mAh/cm^2	2.09 g/cc	161.4 / 160.5 mAh/g	99.4 %	140.7 / 139.9 mAh/g	99.4 %	85.9 %
**Wet 2**	34.26 mg/cm^2	5.42 mAh/cm^2	2.08 g/cc	160.7 / 158.7 mAh/g	98.8 %	146.0 / 145.5 mAh/g	99.6 %	91.4 %
**Wet 3**	33.42 mg/cm^2	5.28 mAh/cm^2	2.09 g/cc	160.6 / 158.0 mAh/g	98.4 %	138.5 / 137.7 mAh/g	99.4 %	87.2 %
**Wet 4**	33.23 mg/cm^2	5.25 mAh/cm^2	2.15 g/cc	160.8 / 158.5 mAh/g	98.6 %	143.8 / 143.2 mAh/g	99.6 %	90.3 %
**Wet 5**	33.34 mg/cm^2	5.27 mAh/cm^2	2.10 g/cc	161.1 / 160.5 mAh/g	99.6 %	142.2 / 141.6 mAh/g	99.6 %	88.2 %
**Dry 1**	32.94 mg/cm^2	5.21 mAh/cm^2	2.3 g/cc	161.3 / 159.6 mAh/g	98.9 %	157.9 / 156.8 mAh/g	99.3 %	98.2 %
**Dry 2**	32.86 mg/cm^2	5.20 mAh/cm^2	2.31 g/cc	161.4 / 160.2 mAh/g	99.3 %	158.2 / 157.3 mAh/g	99.4 %	98.0 %
**Dry 3**	33.6 mg/cm^2	5.31 mAh/cm^2	2.29 g/cc	160.6 / 159.2 mAh/g	99.1 %	154.6 / 153.7 mAh/g	99.4 %	96.5 %
**Dry 4**	33.47 mg/cm^2	5.29 mAh/cm^2	2.32 g/cc	162.2 / 161.6 mAh/g	99.6 %	159.6 / 158.4 mAh/g	99.2 %	98.0%
**Dry 5**	33.46 mg/cm^2	5.29 mAh/cm^2	2.3 g/cc	161.3 / 160.4 mAh/g	99.4 %	157.8 / 156.5 mAh/g	99.2 %	97.6 %

Table S1. The table of information about the tested LFP-Li cells in our work.

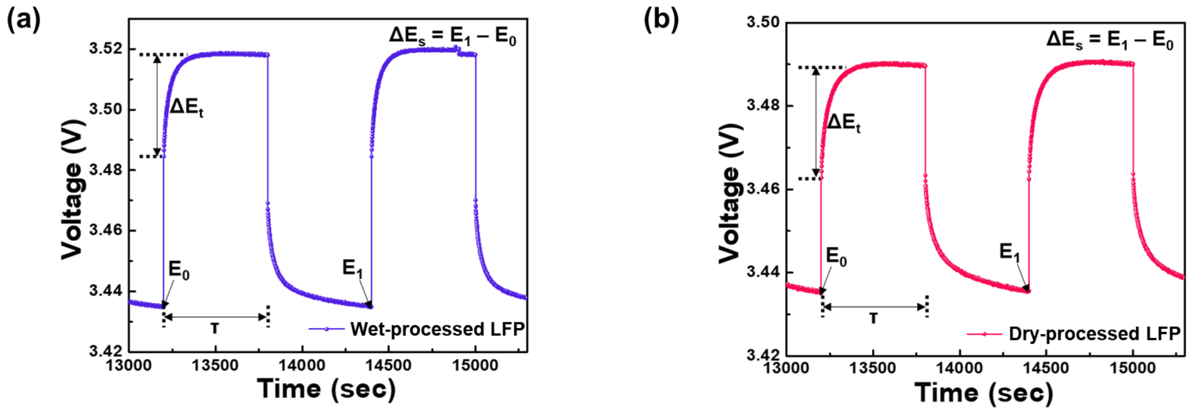

Figure S3. Calculation of D_{Li^+} of (a) wet- and (b) dry-processed LFP electrode through the GITT analysis. The D_{Li^+} can be calculated by using the following equation [1].

$$D_{Li^+} = \frac{4}{\pi\tau}\left(\frac{m_B V_M}{M_B S}\right)^2 \left(\frac{\Delta E_S}{\Delta E_t}\right)^2 \qquad (1)$$

V_m is the molar volume of LFP

S is the specific active surface area of the electrode

m_B and M_B are the molecular weight and mass of the active material, respectively

τ is the duration of the current pulse

ΔE_s is the potential in the steady state

ΔE_t is the potential in the charging/discharging step

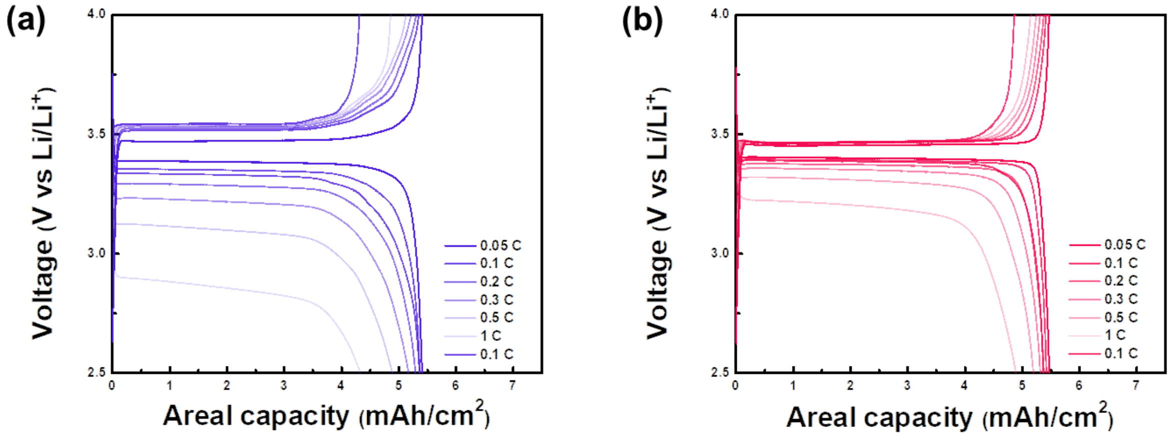

Figure S4. The voltage profiles of LFP-Li cells with (a) wet- and (b) dry-processed LFP electrode from 0.05C to 1C.

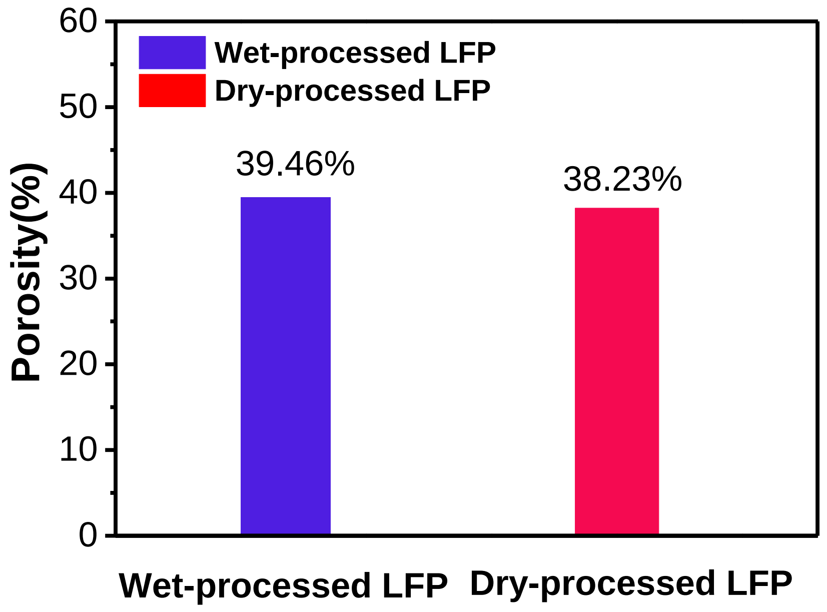

Figure S5. The porosity of wet- and dry-processed LFP electrodes by mercury porosimetry measurement**.**

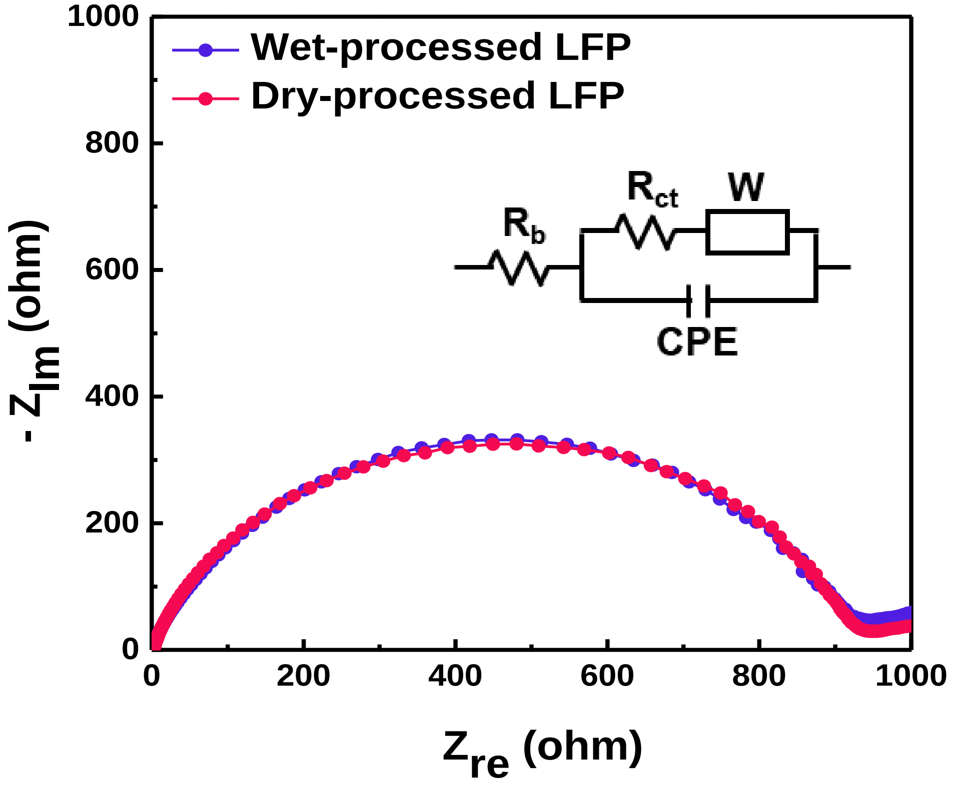

Figure S6. The EIS spectra of wet- and dry-processed LFP electrode before cycle.

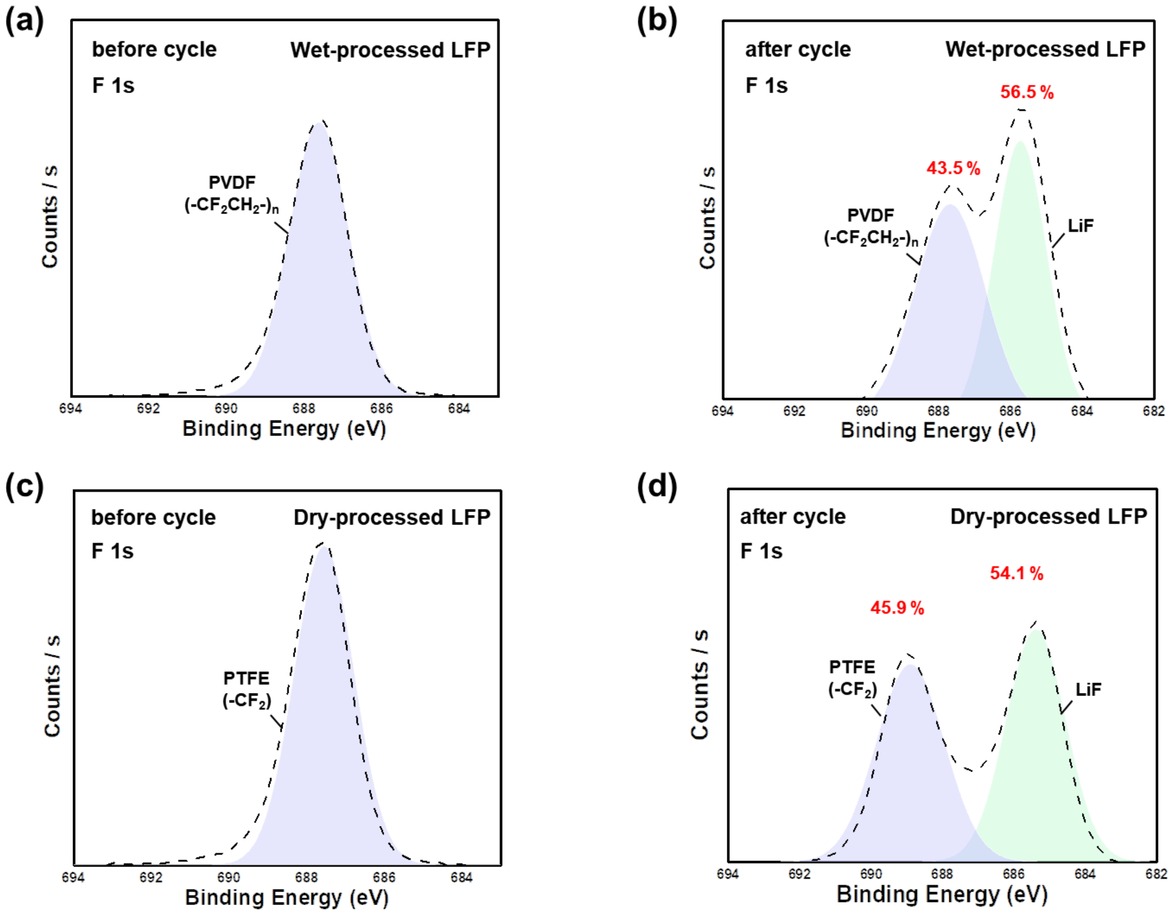

Figure S7. F 1S XPS spectra of (a, b) wet- and (c, d) dry-processed electrodes before and after cycle.

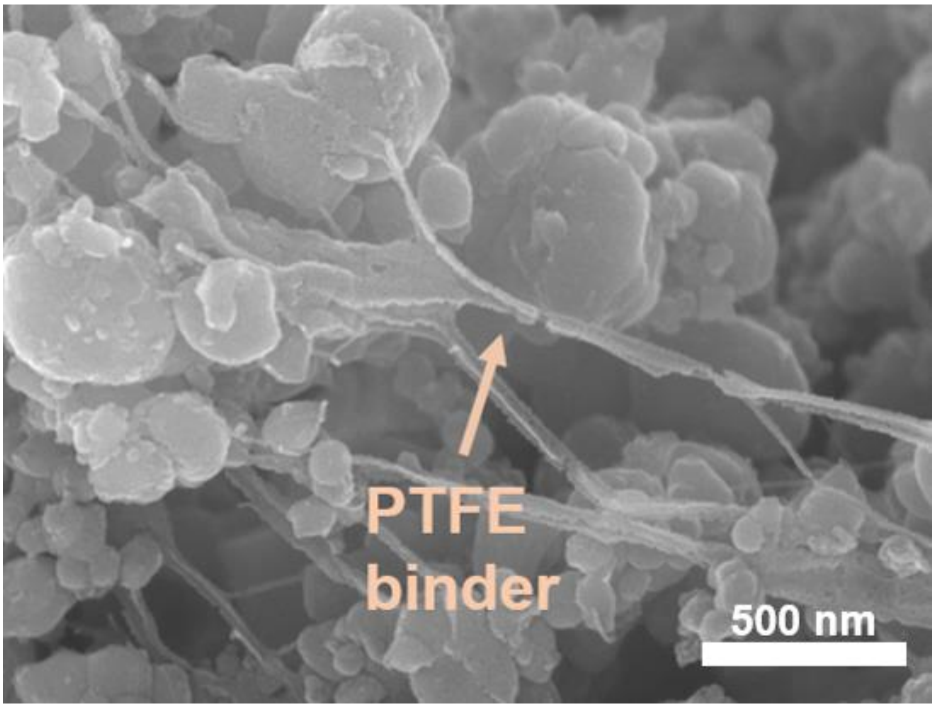

Figure S8. SEM images of PTFE fiber in the dry-processed LFP electrode after cycles.

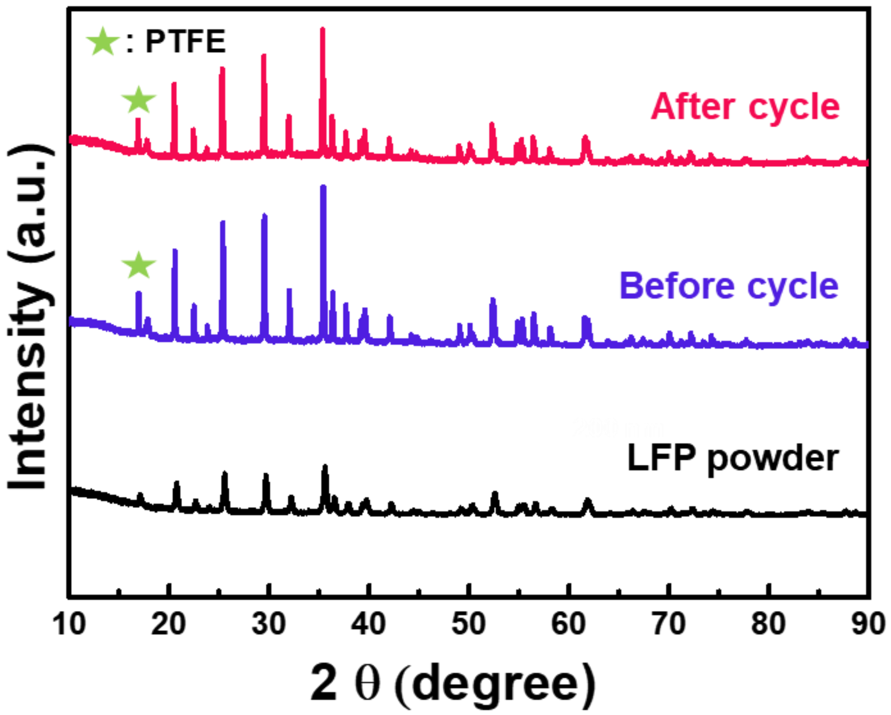

Figure S9. The XRD patterns of LFP powder and dry-processed LFP electrode before and after cycle.

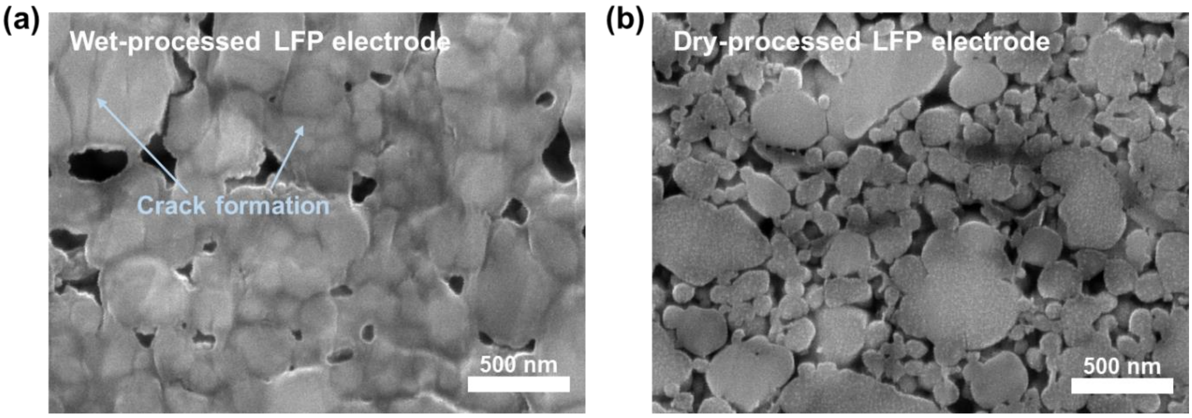

Figure S10. Cross-sectional SEM images of wet-processed and dry-processed LFP electrodes after cycling.

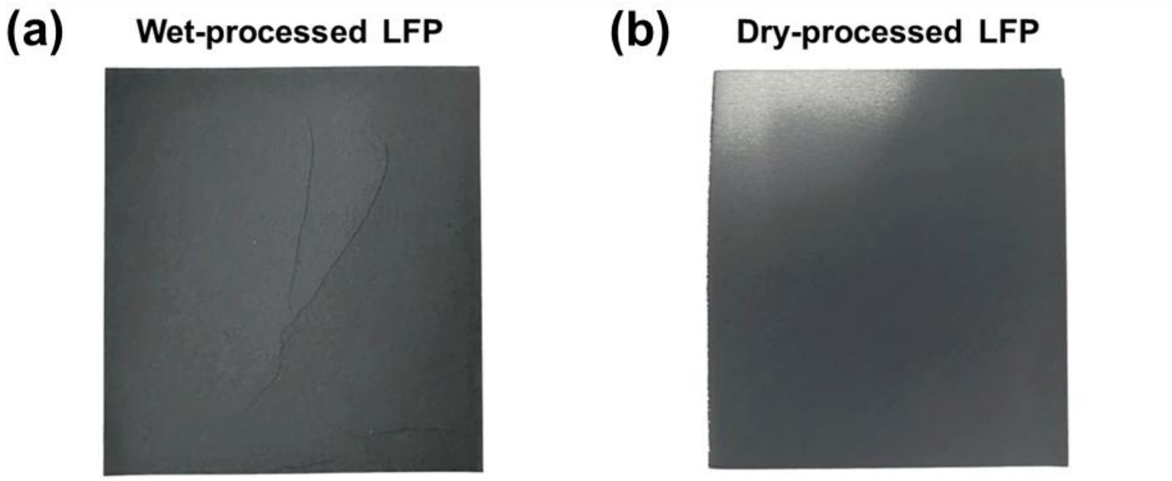

Figure S11. The digital images of (a) wet- and (b) dry-processed LFP electrode with high mass loading (7.8 mAh cm^{-2}).

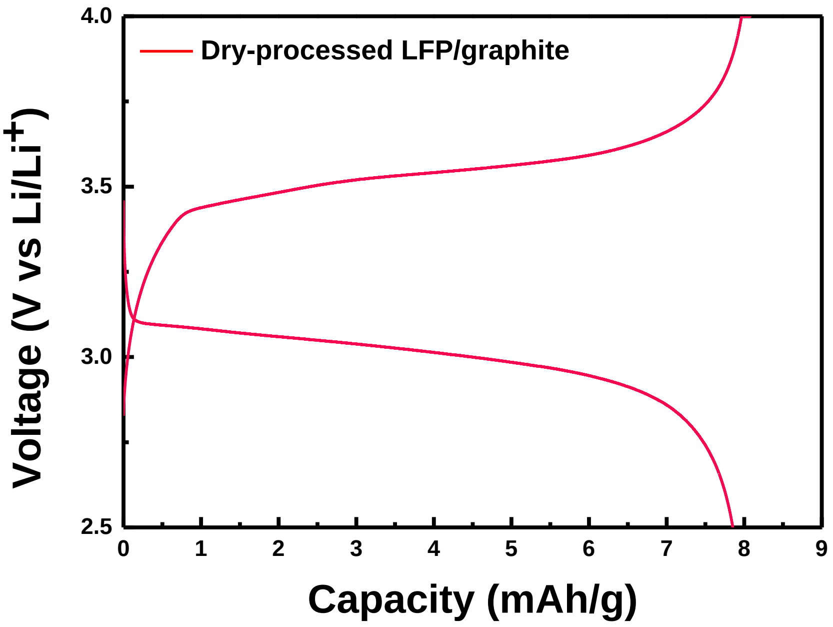

Figure S12. The voltage profiles of dry-processed LFP/graphite full cell at 1st cycle.

Table S2. The table of information about the LFP cathode in our work and previous literature.

Ref	Areal capacity (mAh cm^{-2})	Volumetric capacity (cathode, mAh cm^{-3})	Composition ratio (LFP ratio)	Density (g/cc)	Methods
This work	7.8	376.8	97	2.3	Dry
[2]	4.56	141.9	80	0.91	Wet
[3]	0.83	118.57	70	1	Wet
[4]	3.2	248	80	1.55	Wet
[5]	3.1	256.2	90	2.0	Dry
[6]	3.14	129.2	37.43	2.2	Wet
[7]	2.53	314	80.8	1.965	Wet
[8]	2.76	168	80	-	Wet
[9]	2.42	234.35	80	1.38	Wet
[10]	1.5	68.18	76	1.9	Cold plasma coating

Table S3. Estimated energy density of prismatic cell for electric vehicle.

	Wet Electrode (Thin)	Wet Electrode (Thick)	Dry Electrode (Super Thick)	Dry Electrode + High Density LFP
**Electrode Technology**	Wet		Dry	
**Cathode Areal Capacity**	3 mAh/cm^2	5 mAh/cm^2	7.8 mAh/cm^2	
**Loading Level**	20 mg/cm^2	33 mg/cm^2	52 mg/cm^2	
**Gravimetric Energy Density**	118 Wh/kg	153 Wh/kg	185 Wh/kg	
**Volumetric Energy Density**	375 Wh/L	390 Wh/L	400 Wh/L	470 Wh/L

Reference

[1] A. Nickol, T. Schied, C. Heubner, M. Schneider, A. Michaelis, M. Bobeth, G. Cuniberti, GITT analysis of lithium insertion cathodes for determining the lithium diffusion coefficient at low temperature: challenges and pitfalls, Journal of The Electrochemical Society, 167 (2020) 090546.
[2] Y. Zhang, F. Li, K. Yang, X. Liu, Y. Chen, Z. Lao, K. Mai, Z. Zhang, Polymer Molecular Engineering Enables Rapid Electron/Ion Transport in Ultra-Thick Electrode for High-Energy-Density Flexible Lithium-Ion Battery, Advanced Functional Materials, 31 (2021) 2100434.
[3] Y. Chen, K. Fu, S. Zhu, W. Luo, Y. Wang, Y. Li, E. Hitz, Y. Yao, J. Dai, J. Wan, V.A. Danner, T. Li, L. Hu, Reduced Graphene Oxide Films with Ultrahigh Conductivity as Li-Ion Battery Current Collectors, Nano Letters, 16 (2016) 3616-3623.
[4] Y. Kuang, C. Chen, G. Pastel, Y. Li, J. Song, R. Mi, W. Kong, B. Liu, Y. Jiang, K. Yang, L. Hu, Conductive Cellulose Nanofiber Enabled Thick Electrode for Compact and Flexible Energy Storage Devices, Advanced Energy Materials, 8 (2018) 1802398.
[5] Y. Zhang, S. Lu, F. Lou, Z. Yu, Solvent-free lithium iron phosphate cathode fabrication with fibrillation of polytetrafluoroethylene, Electrochimica Acta, 456 (2023) 142469.
[6] J. Wang, M. Wang, N. Ren, J. Dong, Y. Li, C. Chen, High-areal-capacity thick cathode with vertically-aligned micro-channels for advanced lithium ion batteries, Energy Storage Materials, 39 (2021) 287-293.
[7] H. Zheng, J. Li, X. Song, G. Liu, V.S. Battaglia, A comprehensive understanding of electrode thickness effects on the electrochemical performances of Li-ion battery cathodes, Electrochimica Acta, 71 (2012) 258-265.
[8] Z. Zhao, M. Sun, W. Chen, Y. Liu, L. Zhang, N. Dongfang, Y. Ruan, J. Zhang, P. Wang, L. Dong, Y. Xia, H. Lu, Sandwich, Vertical-Channeled Thick Electrodes with High Rate and Cycle Performance, Advanced Functional Materials, 29 (2019) 1809196.
[9] Q. Wang, Z. Chen, X. Zhang, C. Lin, Y. Feng, Y. Zhang, Structural engineering for double-layer high-load LiFePO4 electrode with vertical imparity distribution of conductive additives, Journal of Power Sources, 527 (2022) 231106.
[10] Z. Liang, T. Li, H. Chi, J. Ziegelbauer, K. Sun, M. Wang, W. Zhang, T. Liu, Y.-T. Cheng, Z. Chen, X. Gayden, C. Ban, Solvent-Free Manufacturing of Lithium-Ion Battery Electrodes via Cold Plasma, ENERGY & ENVIRONMENTAL MATERIALS, n/a (2022) e12503.
